# Delayed blood arrival partially mediates the association between arterial dilatation and cerebral small vessel disease

**DOI:** 10.1002/alz.093459

**Published:** 2025-01-09

**Authors:** W Hudson Robb, Panpan Zhang, Krish U Shah, Michelle L. Houston, Niranjana Shashikumar, Niranjana Shashikumar, Kimberly R. Pechman, Dandan Liu, L. Taylor Davis, Angela L. Jefferson, Timothy J. Hohman

**Affiliations:** ^1^ Vanderbilt Memory & Alzheimer’s Center, Vanderbilt University Medical Center, Nashville, TN USA; ^2^ Department of Biostatistics, Vanderbilt University Medical Center, Nashville, TN USA; ^3^ Department of Neurology, Vanderbilt University Medical Center, Nashville, TN USA

## Abstract

**Background:**

Cerebral arterial dilatation, signifying outward vascular remodeling, is linked to a higher risk of Alzheimer’s disease and a higher burden of white matter hyperintensities (WMH). Arterial dilatation may disrupt cerebral hemodynamics and lead to delayed blood arrival to the brain, which is itself linked to an increased burden of WMH. We examined if arterial dilatation was associated with blood arrival timing and if blood arrival timing mediated the effect of arterial dilatation on WMH burden.

**Methods:**

Vanderbilt Memory and Aging Project participants free of clinical dementia or stroke at enrollment (n=249, 73±8 years, 85% cognitively unimpaired, 41% female) underwent multimodal 3T brain magnetic resonance imaging. Internal carotid artery (ICA) and basilar artery (BA) arterial inner diameters were quantified by an expert rater using vessel wall imaging. An established marker of blood arrival timing, the arterial spin labeling spatial coefficient of variation (ASL‐sCoV), was quantified using pseudo‐continuous ASL. Higher ASL‐sCoV indicates slower blood arrival. WMH burden was quantified using semi‐automated methods. Linear regressions related ICA and BA inner diameters to ASL‐sCoV and log‐transformed WMH burden in anterior and posterior arterial territories, individually. Models were adjusted for age, sex, race/ethnicity, cognitive status, Framingham Stroke Risk Profile, intracranial volume, and apolipoprotein E‐ε4 status. We also assessed if ASL‐sCoV mediated associations between arterial diameters and WMH burden.

**Results:**

Larger ICA inner diameter was associated with higher anterior ASL‐sCoV (p=1.7x10^‐8^) and higher anterior WMH burden (p=9.6x10^‐5^). Anterior ASL‐sCoV partially mediated the association between ICA inner diameter and anterior WMH burden (proportion mediated=15%, p=0.04). Larger BA inner diameter was associated with higher posterior ASL‐sCoV (p=1.3x10^‐8^) but not with higher posterior WMH burden (p=0.06).

**Conclusions:**

Our study illustrates novel links between cerebral vascular structure, cerebrovascular hemodynamics, and vascular brain pathology. Because most of the effect of arterial dilatation on WMH burden was not mediated through blood arrival timing, future work will consider other potential mediators, such as arterial stiffness.

